# Male allocation to ejaculation and mating effort imposes different life history trade-offs

**DOI:** 10.1371/journal.pbio.3002519

**Published:** 2024-05-24

**Authors:** Meng-Han Joseph Chung, Rebecca J. Fox, Michael D. Jennions

**Affiliations:** 1 Division of Ecology and Evolution, Research School of Biology, Australian National University, Canberra, Australia; 2 Stellenbosch Institute for Advanced Study (STIAS), Wallenberg Research Centre at Stellenbosch University, Stellenbosch, South Africa; CNRS Life Sciences and University of Lyon, FRANCE

## Abstract

When males compete, sexual selection favors reproductive traits that increase their mating or fertilization success (pre- and postcopulatory sexual selection). It is assumed that males face a trade-off between these 2 types of sexual traits because they both draw from the same pool of resources. Consequently, allocation into mate acquisition or ejaculation should create similar trade-offs with other key life history traits. Tests of these assumptions are exceedingly rare. Males only ejaculate after they mate, and the costs of ejaculation are therefore highly confounded with those of mating effort. Consequently, little is known about how each component of reproductive allocation affects a male’s future performance. Here, we ran an experiment using a novel technique to distinguish the life history costs of mating effort and ejaculation for mosquitofish (*Gambusia holbrooki*). We compared manipulated males (mate without ejaculation), control males (mate and ejaculate), and naïve males (neither mate nor ejaculate) continuously housed with a female and 2 rival males. We assessed their growth, somatic maintenance, mating and fighting behavior, and sperm traits after 8 and 16 weeks. Past mating effort significantly lowered a male’s future mating effort and growth, but not his sperm production, while past sperm release significantly lowered a male’s future ejaculate quantity, but not his mating effort. Immune response was the only trait impacted by both past mating effort and past ejaculation. These findings challenge the assumption that male reproductive allocation draws from a common pool of resources to generate similar life history costs later in life. Instead, we provide clear evidence that allocation into traits under pre- and postcopulatory sexual selection have different trait-specific effects on subsequent male reproductive performance.

## Abstract 2

Theory assumes that allocation to pre- and post-copulatory sexual traits draws from a common pool of resources, creating equivalent life-history trade-offs. However, this study uses a novel experiment with mosquitofish to reveal independent costs of mating effort and sperm allocation on subsequent male performance.

## Introduction

Accounting for variation in life histories among individuals, populations or species is a central challenge in evolutionary biology [1–3]. A key to understand this variation is to recognize that individuals have limited resources to allocate to traits. Time, material or energy allocated to one trait often lowers its availability for another. This creates life history trade-offs such that fitness is maximized by the optimal allocation of resources. Notably, allocation by males into sexually selected traits that increase mating success lowers allocation into naturally selected traits or somatic maintenance [4–6]. Sexually selected traits such as extravagant ornaments or weapons, combat with rivals, or engaging in courtship often elevate mortality [7–9] because they are energetically expensive [10] or activate physiological pathways with harmful pleiotropic effects on somatic maintenance (e.g., immunosuppression; [11,12]). Far less is known about the life history costs of traits under postcopulatory sexual selection that increase fertilization success when females mate multiply and sperm compete (e.g., larger testes, bigger ejaculates; [13,14]). Although it is well established that “sperm is not cheap” [15–17], the life history costs of increased ejaculate allocation under competition are often unclear [18].

Theoretical models of sperm competition games predict a direct trade-off between traits that enhance mate acquisition and traits that elevate sperm competitiveness based on the assumption that both types of traits draw from a single, common pool of resources [19–21]. In support of this assumption, some comparative studies report a negative relationship between pre- and postcopulatory sexual traits after controlling for confounding factors that affect the benefits of allocation (e.g., how readily females are monopolized by males; [22]). However, not all traits under precopulatory sexual selection seem to trade off with ejaculate traits (e.g., ornaments do, but weapons do not, in primates; [23]), and a recent meta-analysis has failed to find any genetic correlation between nonsurvival related traits, including fertility [24]. Basic physiology provides an obvious reason to challenge the assumption that allocation into ejaculates and mating effort is equivalent. Sperm production depends heavily on meiosis in germline tissues [25], while mate acquisition depends on allocation in somatic tissues maintained by mitosis (e.g., the production of physical traits or the use of muscles in sexual displays or combat; [26,27]). This raises the issue of whether it is appropriate to assume that ejaculates and mating effort draw from a single pool of resources. Predictions about life history trade-offs change if ejaculates and mating effort impose different costs. For example, if the costs of guarding females (i.e., greater mating costs with lower ejaculate expenditure) differ from those of sneaking copulation (i.e., lower mating costs with greater ejaculate expenditure) [28], then changes in social status (e.g., switching between guarding and sneaking) can alter how early-life reproduction diverts energy from late-life performance [3], resulting in a range of possible patterns of male senescence.

To date, very few studies have used an experiment to compare the effect of pre- versus postcopulatory sexually selected traits on life history trade-offs. These trade-offs cannot be reliably detected by comparing mated and virgin individuals [29–31]: Mated males who ejaculate (and then replenish sperm) have already paid the preceding costs of mate acquisition. More generally, males with greater precopulatory allocation (i.e., larger sexual ornaments, higher courtship rate) have greater mating success, which, in turn, results in more frequent sperm replenishment [32]. The costs of mating effort and ejaculation are therefore highly confounded and difficult to separate statistically. Ultimately, an experimental approach is essential to determine the causal effects of mating effort and ejaculation on each other and on other traits. However, such experiments are lacking due to the logistic challenge of manipulating the ejaculate rate without changing the mating rate.

We have developed a surgical technique for mosquitofish, *Gambusia holbrooki*, which provides a way to address the question of the relative life history costs of ejaculation and mating effort [33]: We prevent males from ejaculating by ablating the tip of their intromittent organ. Ablated males behave normally and try to mate, but they do not receive the sensory cues that induce sperm release [33]. Surgery by itself does not affect male attractiveness [34], male–male aggression (this study), or baseline sperm production [35]. Male *G*. *holbrooki* attempt to mate every few minutes [36]; and females mate multiply [37], which selects for large ejaculates [14]. Ejaculate production seems costly as greater food availability increases ejaculate size and hastens sperm replenishment [38]. Strong competition for mates and high sperm competition make *G*. *holbrooki* an ideal species to study the costs of male allocation into sexually selected traits.

We randomly assigned males to 3 treatments to independently manipulate mating effort and the frequency of sperm release/replenishment. To quantify the cost of mating effort, we compared (a) *naïve* males without access to females and rivals that neither attempted to mate nor ejaculated, to (b) *ablated* males with access to females and male rivals that could mate/fight but not ejaculate. To quantify the cost of sperm release/replenishment, we compared (b) *ablated* males to (c) *non-ablated* males with access to females and rivals that could both mate/fight *and* ejaculate. To quantify trade-offs, we measured both somatic traits (growth, immune response) and reproductive traits (mating and aggressive behavior, sperm traits) after 8 and 16 weeks. Mating and fighting behavior were measured under standardized conditions: the focal male was placed in a tank containing a new female and a novel rival male for behavioral observation. Ejaculates were also measured under standardized conditions: to control for treatment-induced variation in a male’s recent history of mating and ejaculation, the focal male was stripped of sperm and isolated for 5 days [38] before we stripped his sperm again to quantify the accumulated sperm reserves (*total sperm count* and *sperm velocity*); after 24 hours, we again stripped sperm from the male to measure his *rate of sperm replenishment*.

If mating effort and ejaculation draw on the same (or a highly overlapping) pool of resources, all else being equal, we predict that:

1. Increased mating effort will lower future sperm production, and vice versa.
2. Mating effort and ejaculation will affect the same naturally selected traits.
3. The costs of mating effort and ejaculation will cumulatively increase and be more apparent in older males.

Our current study extends an earlier study [34] in 2 key ways. First, all focal males were housed with a female and 2 rival males to provide social cues about sexual competition. In the earlier study, focal males lacked rivals and social cues about sexual competition. Their absence might have lowered reproductive allocation, making it harder to detect life history trade-offs. Second, we measured costs at 16 weeks. Previously we only measured costs after 8 weeks [34]. Adult males can live up to 20 weeks in the wild [39]. In our current study, we can therefore test for costs of reproductive allocation that might be absent in younger males. In general, the costs of reproduction accelerate with age [3,40].

## Results

### Preliminary test: No surgery effect on performance during male–male interactions

To test whether the ablation surgery itself causes behavioral changes in the current experiment where males compete with rivals, we haphazardly assigned virgin males to be either *ablated* or *non-ablated* (two-sample *t* test for body size: *t* = 0.517, *p* = .606; *n* = 70 per group). We measured mating behavior before and after each male was housed with a female and 2 rival males for 7 days of sexual interactions. Previous studies have shown that 7 days suffices to detect behavioral adjustments in response to past sperm allocation [41] or past mating effort [42].

There was no detectable effect of ablation surgery on male behavior (Table 1), except that *ablated* males made fewer mating attempts on Day 0. However, this effect was undetectable after 7 days (Table 1), indicating no long-term effects of surgery.

**Table 1:** Surgery effect (i.e., gonopodium ablated or intact) on mating behaviors.

Trait	Predictor	Test statistic	*p*
**Number of nips** and approaches	Body length * Day	*χ²*_1_ = 4.248	**.039**
	Gonopodium state	*χ²*_1_ = 0.282	.596
	Day	*χ²*_1_ = 47.419	**< .001**
	Body length (standardized)	*χ²*_1_ = 2.965	.085
	Male size difference (standardized)	*χ²*_1_ = 7.625	**.006**
**Time spent near female**	Gonopodium state	*F*_1,139.06_ = 0.220	.639
	Day	*F*_1,134.21_ = 0.767	.383
	Body length (standardized)	*F*_1,188.74_ = 5.010	**.026**
	Male size difference (standardized)	*F*_1,238.52_ = 0.332	.565
**No. mating attempts**	Gonopodium state * Day	*χ²*_1_ = 5.838	**.016**
	Gonopodium state	*χ²*_1_ = 2.372	.124
	Day	*χ²*_1_ = 33.468	**< .001**
	Body length (standardized)	*χ²*_1_ < 0.001	.996
	Male size difference (standardized)	*χ²*_1_ = 1.460	.227
First day of sexual interaction (Day 0)	Gonopodium state * Body length	*χ²*_1_ = 0.028	.867
	Gonopodium state	*χ²*_1_ = 5.030	**.025**
	Body length (standardized)	*χ²*_1_ = 0.135	.713
	Male size difference (standardized)	*χ²*_1_ = 0.741	.389
Seven days after sexual interaction (Day 7)	Gonopodium state * Body length	*χ²*_1_ = 0.093	.761
	Gonopodium state	*χ²*_1_ = 1.812	.178
	Body length (standardized)	*χ²*_1_ = 0.238	.625
	Male size difference (standardized)	*χ²*_1_ = 3.428	.064

Given the significant interaction between gonopodium state and day on *number of mating attempts*, the surgery effect was tested separately on each day. Statistical outputs of final models excluding nonsignificant interactions are shown. Full models are provided in Table A in S1 Text. The bold font highlights significance at the 0.05 level.

### The effect of past reproductive allocation on somatic traits

**Fig 1:**
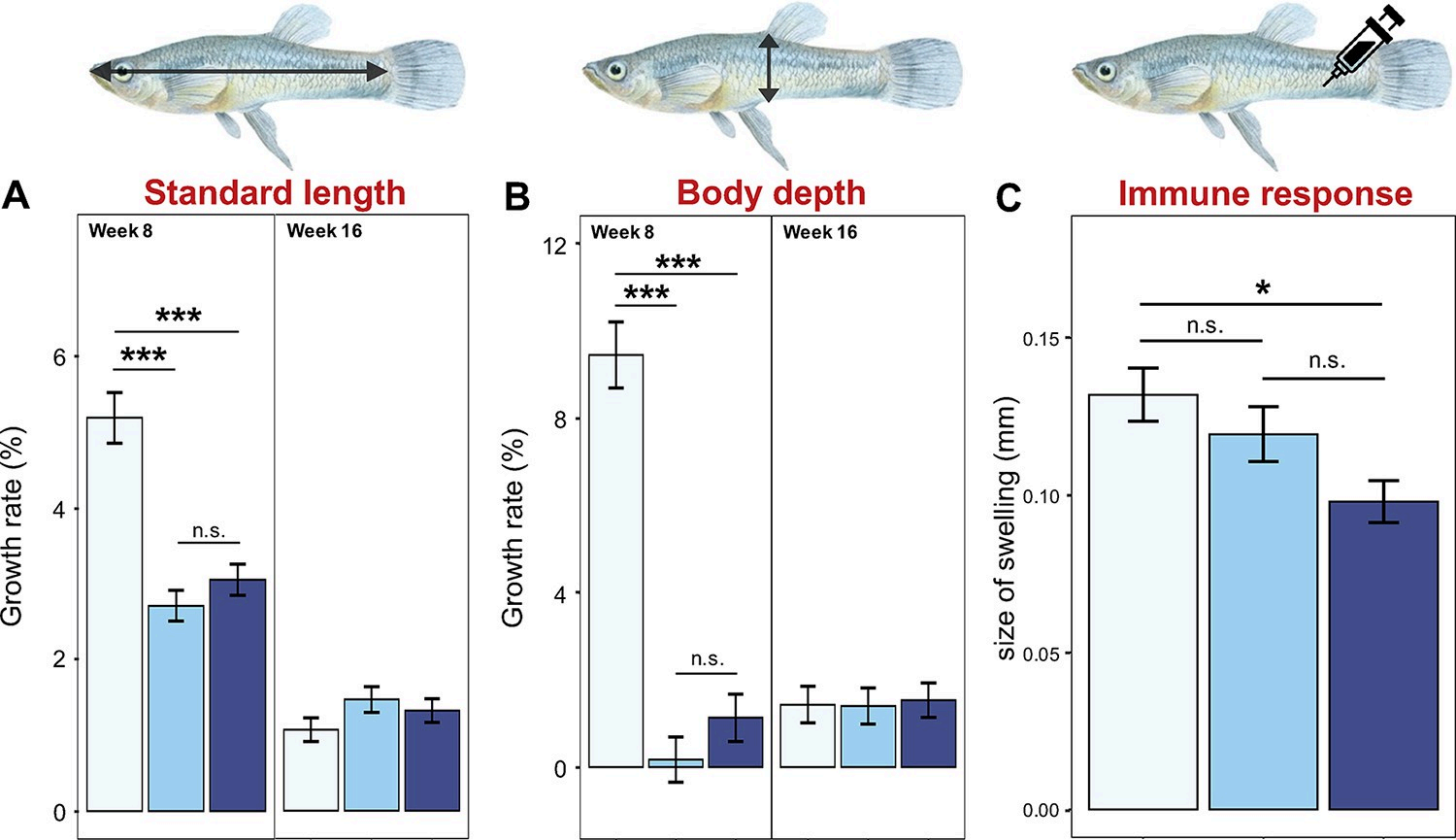
Effects of reproductive treatment and age at testing on somatic traits. (**A**) Standard length, (**B**) body depth, and (**C**) immune response. When there is a significant treatment * age interaction (see Table B in S1 Text for details), data at weeks 8 and 16 are presented separately. Light blue = *naïve* males that neither mated nor ejaculated; blue = *ablated* males that mated but did not ejaculate (hence low/no sperm replenishment); dark blue = *non-ablated* males that both mated and ejaculated. Significant level: *** *p* < .001; ** *p* = .001 to .01; * *p* = .01 to .05; n.s. = nonsignificant. *p*-values are from Tukey’s tests (see text). Data are shown as mean ± SE. The data and code used to generate this figure can be found in https://data.mendeley.com/datasets/jv3dyxndtz/1.

Controlling for initial body size, males who had invested in mating effort (i.e., *ablated* or *non-ablated* males) were significantly shorter and thinner at week 8 (Fig 1A and 1B), measured as either standard length (SL; snout tip to base of caudal fin) or body depth (BD; base of dorsal fin to ventral side of body) (Tukey’s tests: versus *naïve* males; all *p* < .001; Table B in S1 Text). In contrast, whether a male released sperm in the first 8 weeks did not affect his SL or BD (Tukey’s tests: *ablated* versus *non-ablated* males, both *p* > 0.25). Males barely grew from weeks 8 to 16 (Fig 1); hence, there was no effect of past reproductive allocation on body size (Table B in S1 Text).

Past reproductive allocation significantly affected immune response (Table B in S1 Text). *Naïve* males had greater immunocompetence than *non-ablated* males, who had invested into both mating effort and ejaculation (Tukey’s test: *p* = .013; Fig 1C). However, neither greater mating effort alone (*naïve* versus *ablated* males) nor frequent sperm release alone (*ablated* versus *non-ablated* males) lowered immunocompetence (Tukey’s tests: both *p* > .165). These treatment effects did not differ between weeks 8 and 16 (interaction: *p* = .538) nor did immune function decline with age (*p* = .401) (Table B in S1 Text).

### The effect of past reproductive allocation on reproductive traits

**Fig 2:**
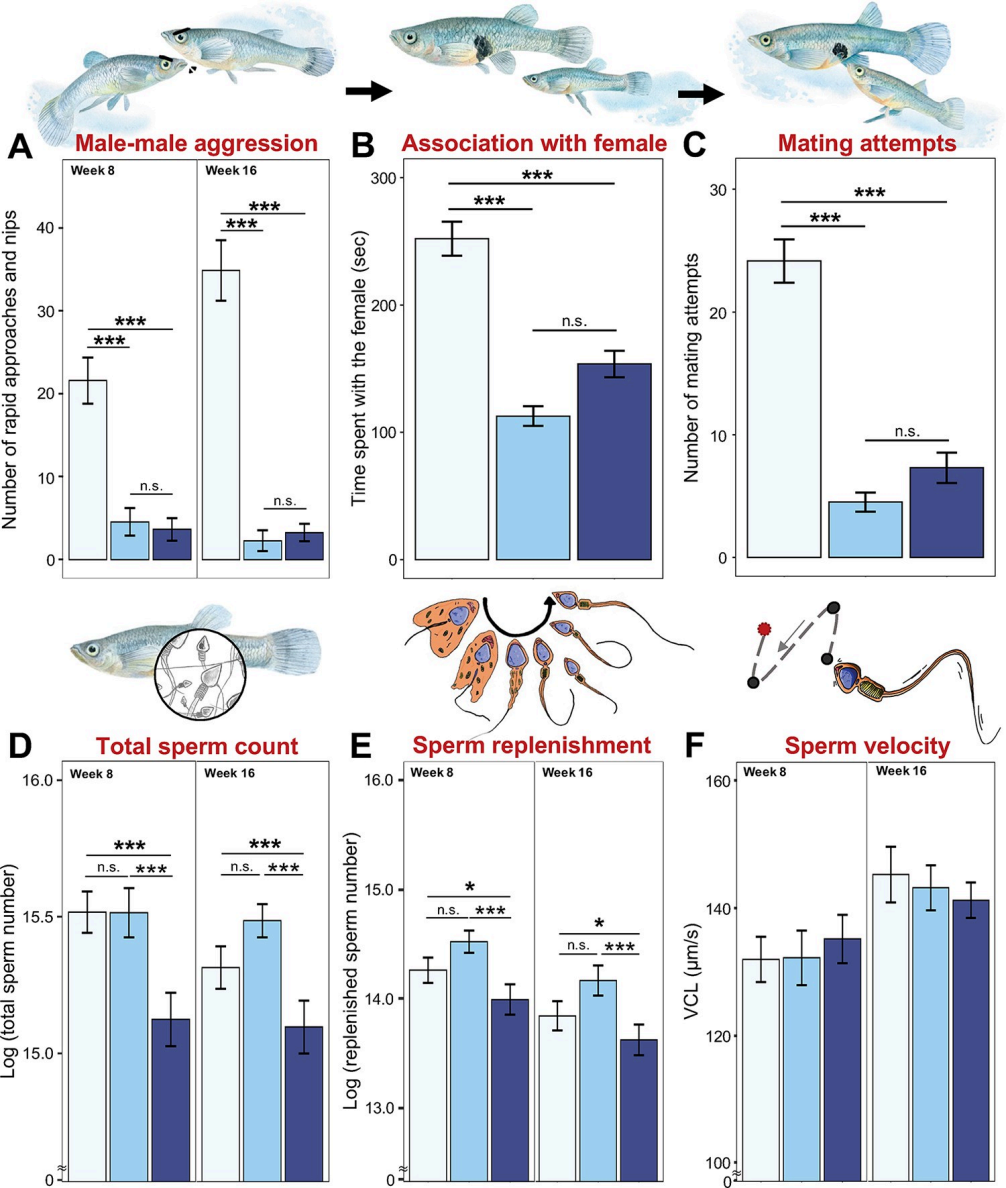
Effects of reproductive treatment and age at testing on reproductive traits. Mating performance: (**A**) number of rapid approach and nips to rival, (**B**) time spent near female, (**C**) number of mating attempts; and ejaculate traits: (**D**) total sperm number (log-transformed), (**E**) number of replenished sperm (log-transformed), (**F**) sperm velocity. Given a significant effect of age (see Table 2 for details), data at weeks 8 and 16 are presented separately. Light blue = *naïve* males; blue = *ablated* males; dark blue = *non-ablated* males. Significant level: *** *p* < .001; ** *p* = .001 to .01; * *p* = .01 to .05; n.s. = nonsignificant. *p*-Values are from Tukey’s tests (see text). Data are shown as mean ± SE. The data and code used to generate this figure can be found in https://data.mendeley.com/datasets/jv3dyxndtz/1.

We tested how past reproductive allocation affected a male’s subsequent mating performance by observing his behavior toward a female in the presence of a novel rival after 8 and 16 weeks. Past reproductive allocation and age at testing did not interact to affect any of the reproductive traits (Table C in S1 Text), but both factors independently affected male behavior (Table 2). *Naïve* males approached and nipped a rival significantly more often than males with greater past mating effort (i.e., *ablated* or *non-ablated*) (Tukey’s tests: both *p* < .001; Fig 2A), but repeated sperm release in the past did not affect male aggression (Tukey’s test: *ablated* versus *non-ablated* males, *p* = .738). Similarly, *naïve* males spent significantly more time near females than did males with higher past mating effort (Tukey’s tests: both *p* < .001; Fig 2B and Table 2), but past sperm release did not affect the time spent near females (Tukey’s test: *p* = .061). Whether or not a male attempted to mate was unaffected by his past reproductive allocation (Table 2). Of those males that attempted to mate, males with greater past mating effort made significantly fewer attempts (*ablated* or *non-ablated* versus *naïve* males, Tukey’s tests: both *p* < .001; Fig 2C). In contrast, past sperm release did not affect the number of mating attempts (Tukey’s test: *p* = .732). Age at testing had no effect on the number of mating attempts or time spent near females, but older males were significantly more aggressive toward rivals (weeks 8 versus 16, Table 2).

**Table 2:** Effects of reproductive treatment and age at testing on male reproductive traits.

Trait		Predictor	Test statistic	p
***Mating performance***				
**No. nips and approaches**		Reproductive treatment	*χ²*_2_ = 192.298	**< .001**
		Age at testing	*χ²*_1_ = 5.533	**.019**
		Male size difference (standardized)	*χ²*_1_ = 0.668	.414
		Initial body length (standardized)	*χ²*_1_ = 3.150	.076
**Time spent near female**		Reproductive treatment	*F*_2,156.16_ = 40.033	**< .001**
		Age at testing	*F*_1,173.29_ = 0.008	.929
		Male size difference (standardized)	*F*_1,302.82_ = 0.446	.505
		Initial body length (standardized)	*F*_1,231.28_ = 1.725	.190
**No. mating attempts**	Zero-inflation	Reproductive treatment	*χ²*_2_ = 4.916	.086
		Age at testing	*χ²*_1_ = 3.714	.054
		Male size difference (standardized)	*χ²*_1_ = 0.007	.932
		Initial body length (standardized)	*χ²*_1_ = 1.158	.282
	Conditional	Reproductive treatment	*χ²*_2_ = 33.512	**< .001**
		Age at testing	*χ²*_1_ = 0.596	.440
		Male size difference (standardized)	*χ²*_1_ = 3.657	.056
		Initial body length (standardized)	*χ²*_1_ = 6.614	**.010**
***Ejaculate traits***				
**Total sperm count**		Reproductive treatment	*F*_2,152.28_ = 13.731	**< .001**
		Age at testing	*F*_1,176.66_ = 8.587	**.004**
		Initial body length (standardized)	*F*_1,169.44_ = 59.365	**< .001**
**Sperm replenishment rate**		Reproductive treatment	*F*_2,149.77_ = 10.155	**< .001**
		Age at testing	*F*_1,169.65_ = 35.127	**< .001**
		Initial body length (standardized)	*F*_1,164.76_ = 37.206	**< .001**
**Sperm velocity (VCL)**		Reproductive treatment	*F*_2,295_ = 0.061	.941
		Age at testing	*F*_1,295_ = 6.512	**.011**
		Initial body length (standardized)	*F*_1,295_ = 13.370	**< .001**

Main effects are obtained from final models excluding any nonsignificant interactions. Statistical outputs of initial models with the interaction are provided in Table C in S1 Text.

Ejaculate quantity (total sperm count and sperm replenishment rate), but not sperm velocity, was significantly affected by past reproductive allocation (Table 2). In contrast to mating behavior, however, the decline in total sperm count and sperm replenishment rate were due to past sperm release (Tukey’s test: *ablated* versus *non-ablated*, both *p* < .001; Fig 2D and 2E), but not past mating effort (Tukey’s test: *naïve* versus *ablated*, *p* = .661 and .082). Finally, older males had a significantly lower total sperm count and a slower rate of sperm replenishment, but they produced sperm that swam significantly faster (Table 2).

## Discussion

Current reproductive allocation is assumed to impose costs by depleting a common pool of resources that are also used for growth, maintenance, and future reproduction [43–45]. This creates life history trade-offs among different components of fitness. Similarly, there are trade-offs among traits, including ones that affect the same fitness component. Specifically, for sexually selected traits that increase the rate of reproduction, “sperm competition game” models of optimal allocation make the assumption that “*each male has a fixed total energy budget for reproduction (R)*, *which he divides between his allocation to precopulatory competition (T) and his allocation to postcopulatory competition (U)*, *so that*: *R = T + U*” [20,21]. Hence, at any given time, greater mating effort should reduce ejaculate production, and vice versa [19]. This prediction has sometimes been extended to argue for *long-term* trade-offs between pre- and postcopulatory expenditures. For example, male houbara bustards with a higher rate of sexual displays had a more rapid decline in ejaculate viability with age [46]. However, these males would have ejaculated more frequently because females prefer males with greater courtship effort [47]. In observational studies, distinguishing how pre- and postcopulatory reproductive allocation affects life history trade-offs in the long run is therefore challenging because allocation in mate acquisition always precedes ejaculation.

**Fig 3:**
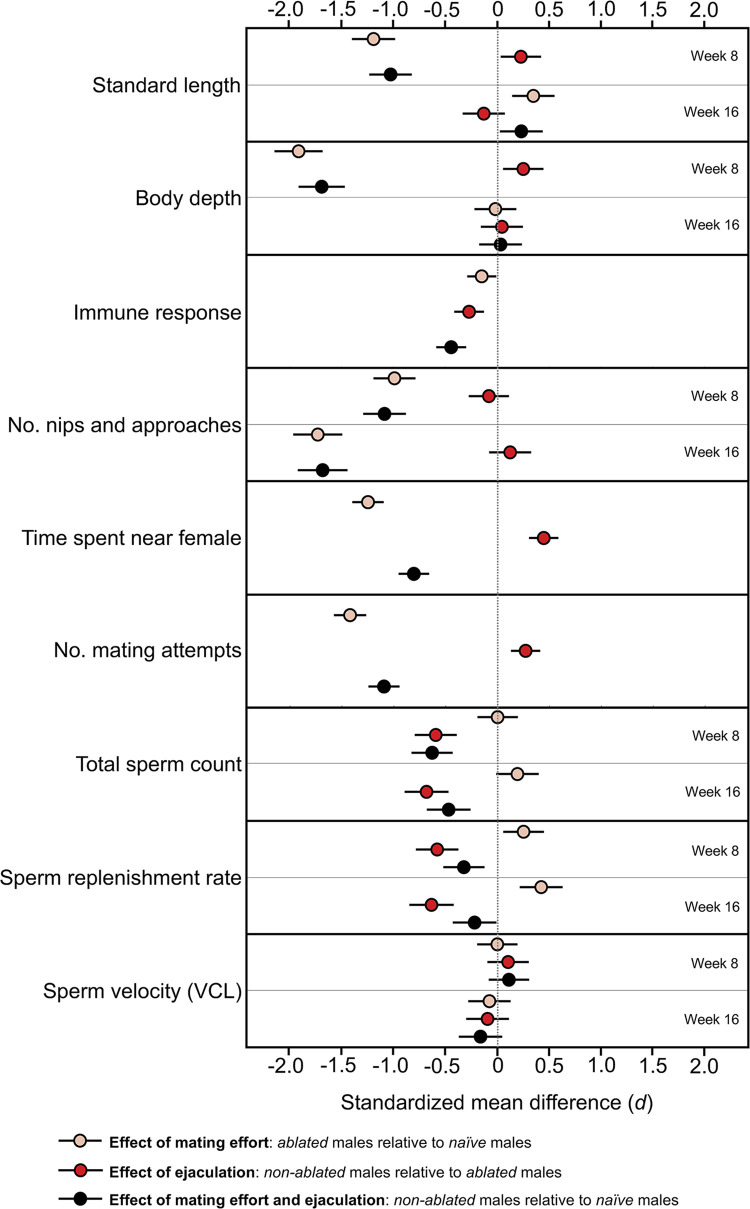
Standardized mean differences (Cohen’s *d*) showing the effect of past mating effort and/or past ejaculation on life history and reproductive traits. Error bars represent mean ± SD (see [51] for calculation). The data and code used to generate this figure can be found in https://data.mendeley.com/datasets/jv3dyxndtz/1.

Our results only reveal that both ejaculation and mating effort jointly imposed a cost on immune response. This represents one of the first pieces of evidence that repeated sperm release/replenishment could impair immunity in the long run (for a short-term trade-off, see [48]). However, for all other traits, we found independent effects of mating effort and ejaculation (Fig 3): Past sperm release only affected future ejaculate production, while past mating effort only affected future mating performance and growth. We can therefore reject prediction (1) that greater mating effort will lower future sperm production, and vice versa; and only partly agree with prediction (2) that mating effort and ejaculation will affect the same naturally selected traits. Our findings offer insights into how to apply life history trade-offs to different sexually selected traits and illuminate hypotheses about differences among traits in their later-life performance [49,50]. For example, greater allocation to ejaculate size due to a higher perceived risk of sperm competition [13] could accelerate the reduction of ejaculate quality with advancing age but have no impact on the courtship or fighting ability of older males.

### Mating effort and sperm release have different effects on later-life performance

Why did past mating effort reduce male growth? First, spending more time pursuing females and attacking rivals reduces the time available to forage. In guppies, males spent 65% to 75% less time foraging when trying to mate [52], and male mosquitofish are likely to experience a similar time trade-off. In addition, physical encounters with a female and rivals might increase the levels of competition for food, which could lower resource acquisition. Second, the expression of sexual behaviors often elevates testosterone levels [53,54], which tends to be associated with a higher metabolic rate and energy consumption [55]. Even so, it is difficult to explain the effect of mating effort on growth solely by invoking energy availability or resource acquisition, as past mating effort did not affect future sperm production, which also has energetic costs [17]. Another explanation why mating effort reduced growth is that prolonged locomotion when chasing females generated reactive oxygen species, which damaged somatic cells and muscles [56]. Damage to somatic tissue could also explain the decline in future mating performance, but not in future sperm production if germline tissue remains unaffected. Another possibility is that ablated males strategically, and plastically, decrease their mating effort because they detected their failure to inseminate females, possibly based on the lack of decline in their sperm reserves. This seems unlikely, however, given our preliminary test showed no short-term decline in mating effort by ablated males that did not ejaculate (Table 1).

Similarly, why did sperm release and replenishment affect future ejaculate production, but not future mating or fighting behavior, nor growth? Following the logic outlined above, this might reflect costly damage that is localized to germline tissue, such as toxic metabolic waste products accumulating in the testes [25], but has no effect on somatic tissue. Our finding that past allocation in ejaculation lowered future sperm quantity, but not sperm velocity, suggests that sperm quantity is more strongly condition-dependent [57]. One possibility is that males benefit from adaptively shifting resources to maintain sperm quality, even if this lowers their sperm count. In support of this explanation, we have shown that, controlling for sperm count, the sperm of mated and virgin male mosquitofish is equally effective for artificial insemination [58], suggesting no difference in sperm quality.

### Age and reproductive decline

Given the importance of reproduction in determining lifetime fitness, one might expect little reproductive decline earlier in adulthood, but a steep decline as males approach their maximum natural life span [59,60]. However, we detected a significant phenotypic decline in most reproductive traits after only 8 weeks of reproductive allocation (Fig 2), indicating that costs arise shortly after males start to engage in reproduction. There were no significant interactions between the reproductive treatment (naive, ablated, non-ablated) and age (weeks 8 or 16) for any of the measured reproductive traits (Table 2), indicating that the main effects of mating effort and ejaculation did not increase for older males. We can therefore reject our prediction (3) that the costs of mating effort and ejaculation will cumulatively increase and be more apparent in older males. Even so, the absolute sperm count and rate of sperm replenishment was generally lower in older males (Fig 2). Conversely, however, older males had faster swimming sperm and were more aggressive to rivals. There are therefore effects of male age itself (or correlates thereof), which are independent of the cumulative effects of mating effort and ejaculation. Our findings indicate that male *G*. *holbrooki* pursue a life history strategy with the potential for high reproductive success early in life, even if this creates intensive wear-and-tear [5,46,61,62].

### Does male–male competition moderate the effects of past reproductive allocation?

To test if male–male competition moderates the observed reproductive costs on later-life performance, we ran an additional set of analyses (S1 Text) to compare a situation when 2 male rivals were present (this study) or absent [34]. In the current study, *ablated* and *non-ablated* males interacted freely with 2 rivals and a female, while *naïve* males had visual access to 2 rivals and a female. In the earlier study [34], focal males were only housed with a female. Data were only available to test for an effect of rivals after 8 weeks.

Male–male competition did not exacerbate the reproductive costs of pursuing females or releasing sperm (Tables D and E in S1 Text). For 7 of the 8 measured traits, there was no interaction between the reproductive treatment and the presence/absence of rivals. In the combined data, greater past mating effort led to slower growth, lower immunocompetence, and fewer mating attempts by *ablated* and *non-ablated* males (Tukey’s tests: versus *naïve* males: all *p* < .013) (Table E in S1 Text), but past sperm release did not have these effects (Tukey’s tests: *ablated* versus *non-ablated*, all *p* > .210). Likewise, past sperm release led to a significantly lower sperm count and sperm replenishment rate in the combined data (Tukey’s tests: *non-ablated* males versus *naïve* or *ablated* males: all *p* < .013), but past mating effort did not have these effects (Tukey’s tests: both *p* > .264; Table E in S1 Text). The sole exception was the time spent near females. In the absence of rivals, past reproductive allocation did not affect the time spent near females [34]. In the presence of rivals, however, males with greater past mating effort (*ablated* or *non-ablated*) spent significantly less time near females than *naïve* males, while past sperm release had no effect (Fig 2B). This suggests that antagonistic interactions with rivals and pursuing females have cumulative costs on a male’s ability to pursue females, perhaps because they both involve locomotion that causes muscle damage.

In sum, there is robust evidence using the data from 2 independent studies that (1) past mating effort reduces growth and lowers immunity; (2) past mating effort and past sperm release lower subsequent reproduction in ways that differ predictably among traits: ejaculation lowers future sperm production, while mating effort lowers future mating performance. Interestingly, antagonistic interactions with rivals did not moderate how past reproductive allocation affected future ejaculate production. As noted earlier, this is consistent with the costs of repeated ejaculation being confined to germline tissue, which would not be directly affected by chasing or fighting rivals. The 2 studies were conducted at different times, albeit using the same population of fish and identical laboratory facilities, so caution is needed when comparing their results. Even so, it is worth noting that males that had previously encountered rivals (i.e., this study) replenished their sperm significantly more rapidly and produced faster sperm (even for *naïve* males that only saw rivals) than males that did not encountered rivals [34] (Table E in S1 Text). This difference is consistent with males adjusting their ejaculate size to the perceived risk/intensity of sperm competition (meta-analysis: [13]).

## Conclusions

We ran an experiment to disentangle how pre- and postcopulatory allocation affects male life history traits when males compete for mates and fertilization. Our key finding is that the long-term costs of mating effort and ejaculation are such that there are no detectable trade-offs between pre- and postcopulatory traits. This finding cautions against the generality of life history models that assume such a trade-off to then predict how males optimize allocation between mate acquisition traits and sperm competitiveness according to, for example, the risk/intensity of sperm competition [20] or a male’s own competitiveness [19]. More generally, social and ecological factors that differentially affect the level of sperm and mating competition can lead to adaptive plastic shifts in reproductive allocation into pre- and postcopulatory traits [5]. This could subsequently generate variation in the rate of senescence of different sexually or naturally selected traits that is not predicted if it is assumed that pre- and postcopulatory allocation in sexual traits has equivalent life history effects.

## Materials and methods

### Origin and maintenance of fish

In May 2020, mosquitofish (*G*. *holbrooki*) were collected from ponds in Canberra, Australia (35° 180 27″ S 149° 070 27.9″ E) (ACT Collection license FS20188) and housed in aquarium facilities at the Australian National University (permit A2021/04). Adults were introduced into single-sex stock 90*l* tanks (40 to 50 fish/aquarium). Juveniles were raised communally until their sex could be determined prior to maturation (an elongated anal fin for males; visible gravid spot for females). Males and females were then separated into single-sex 90*l* tanks to ensure virginity. All fish were maintained under 14:10 L:D photoperiod at 28°C and fed twice daily with *Artemia* nauplii ad libitum and commercial fish flakes if in stock tanks, or only *Artemia* when in individual tanks. The experiment ran from June 2020 to January 2021.

### Experimental design

Male mosquitofish almost exclusively employ a coercive mating strategy: a male approaches a female from behind, swings his gonopodium (intromittent organ) forward, and inserts the tip into her gonopore to transfer sperm [37]. We screened for sexually active males by randomly selecting a virgin male and placing him into a 4*l* tank with a wild-caught female for 5 minutes. Only males who chased the female and attempted to mate were included as focal males. We then placed the male in an ice-slurry for 10 seconds as anesthesia before photographing him to measure his SL and BD. Photographs were analyzed using *ImageJ* [63]. Size-matched males (ANOVA, SL: *F* = 1.228, *p* = .296; BD: *F* = 2.369, *p* = .100) were haphazardly assigned to 3 treatments: (a) “Naïve”: a focal non-ablated virgin male, a wild-caught female, and 2 wild-caught rival males were placed into 3 separate chambers of a 7*l* tank using mesh barriers. The focal male experienced only olfactory and visual cues from the female and the rivals without any physical contact, so he did not fully invest into mating behavior nor did he ejaculate (hence low/no need for sperm replenishment) (*n* = 56); (b) “Mating only” (“ablated male”): A focal ablated virgin male (see below for ablation surgery) was placed with a female and 2 rival males. The focal male engaged in aggressive interactions with the rivals, and attempted to chase and mate the female, but he did not ejaculate (i.e., low/no need for sperm replenishment). He mainly allocated resources to mating effort (*n* = 54); (c) “Mating and ejaculation” (“non-ablated male”): A focal non-ablated virgin male was housed with a female and 2 rivals. The focal male could fight with the rivals, chase the female, and ejaculate, thereby investing into mating effort, ejaculation, and sperm replenishment (*n* = 53). There is evidence that female olfactory cues may moderate life history trade-offs in males [64]. While male mosquitofish are almost always exposed to female olfactory cues in the wild, our study focuses on the effects of physical reproductive encounters (i.e., pursuing females and fighting with rivals) and sperm release, rather than the influence of sensory signals.

Males from all 3 treatments paid the cost of baseline sperm production; however, “mating and ejaculation” *non-ablated* males had to replenish sperm more often due to repeated sperm release each time they mated. This, in turn, promotes a higher division rate in the germline, as well as any maintenance costs of spermatozoa [25]. Although we cannot exclude the possibility that *naïve* or “mating only” *ablated* males discard and/or reabsorb unused sperm, poecilid fish infrequently refresh stored sperm reserves [65]. As such, ejaculate allocation by *naive* and *ablated* males should be far lower than that for *non-ablated* males because *non-ablated* males ejaculated and replenished sperm more often. A comparison between *ablated* and *non-ablated* males is therefore a biologically meaningful estimate of the cost of repeated ejaculation during actual mating.

We anesthetized a focal male using iced water for 10 seconds, placed him on a glass slide, and swung his gonopodium forward under a dissecting microscope. According to his treatment, we either ablated or sham-ablated the tip of his gonopodium with a scalpel blade (Diplomat Blades, Victoria, Australia). The removal of the tip prevents a male from receiving the cues that trigger ejaculation [33]. Males were then transferred into individual 7*l* tanks for a 3-day recovery period, after which we introduced a stimulus female and 2 wild-caught stock males (see above). Focal males were labeled using numbering so that we were blind to their treatment. Stimulus females and male rivals were rotated between tanks weekly to minimize effects of female familiarity and variation in the focal males’ dominance status. To identify the focal male, all rival males were anaesthetized in ice slurry and marked with an elastomer tag (Northwest Marine Technology, Shaw Island, WA) injected subcutaneous below the dorsal fin.

The treatments were maintained for 16 weeks. At the end of weeks 8 and 16, somatic and reproductive traits were measured (see below). Ablated males did not regrow their gonopodium tip (personal observation) nor did gonopodium length increase with age (LM, age at measurement: *F* = 1.582, *p* = .209, with week 0 gonopodium length as a covariate: *F* = 1,331.286, *p* < .001).

### Preliminary test

To test for any potential effects of ablation on male sexual behaviors, we randomly assigned virgin males to be *ablated* or *non-ablated* (*n* = 70 per treatment). After a 3-day recovery (Day 0), we introduced a focal male (ablated or non-ablated), an intact rival male (21.06 ± 0.14 mm SL; *n* = 140) and a wild-caught female (27.98 ± 0.20 mm SL; *n* = 140) into a 7*l* tank. The 3 individuals were separated by 2 mesh barriers for 10 minutes. We then raised the barriers and recorded the following behaviors of the focal male for 20 minutes: (a) number of mating attempts; (b) time spent within 1 SL of the female; (c) number of nips and rapid approaches to the rival male. After the trial, each focal male was housed in a 7*l* tank with a female and 2 rivals for 7 days to allow for sexual interaction (with or without ejaculation depending on his treatment). On Day 7, we reran the behavioral trial using a new rival (21.13 ± 0.13 mm SL; *n* = 130) and a novel female (28.12 ± 0.19 mm SL; *n* = 130) to test for any differences in mating performance. Rivals were marked with an elastomer tag.

### Body growth

Males were photographed under anesthesia to measure their SL and BD after 8 and 16 weeks in the treatments. Growth was determined by their current SL and BD controlling for their previous size.

### Immune response

Cell-mediated immunity was tested using a phytohaemagglutinin injection assay [30]. We used a pressure-sensitive spessimeter (Mitutoyo 547–301, accuracy: 0.01 mm) to measure the body thickness at the posterior end of the dorsal fin of males under anesthesia. We made 5 measures per male and used the average value in our analyses. We then injected 0.01 mg phytohaemagglutinin (dissolved in 10 μl PBS) into the left side of the fish at the point where thickness was quantified. After 24 hours, we remeasured the male and calculated the difference between the pre- and postinjection values as our measure of his immune response.

### Mating performance

After 8 and 16 weeks in their assigned treatments, we examined the effect of reproductive allocation on the focal males’ mating behavior by recording: (a) number of nips and rapid approaches; (b) time spent near the female; and (c) number of mating attempts over 20 minutes following the same protocol used in the Preliminary test.

### Sperm traits

We tested for any differences in sperm traits due to the reproductive treatment after 8 and 16 weeks. To control for variation in sperm age, we first emptied all sperm reserves of focal males (Day 0) and isolated them in separate 7*l* tanks for 5 days to produce new sperm bundles [38]. On Day 5, we put an anaesthetized male on a glass slide covered by 1% polyvinyl alcohol solution, swung the gonopodium forward under a dissecting microscope and gently pressed on his abdomen to eject sperm bundles. We collected the sperm into a known volume (200 to 1,200 μl depending on ejaculate size to optimize the count measurement) of extender medium (207 mM NaCl, 5.4 mM KCl, 1.3 mM CaCl_2_, 0.49 mM MgCl_2_, 0.41 mM MgSO_4_, 10 mM Tris (Cl) (pH 7.5)). After vortexing the solution, we placed 3 μl on a 20-μm capillary slide (Leja) under 100× magnification. Sperm number was calculated using the automated program CEROS Sperm Tracker (Hamilton Thorne Research, Beverly, MA, USA). After 24 hours (Day 6), we again stripped the male and counted the number of sperm as a measure of his daily rate of sperm replenishment. We determined the total sperm count and number of replenished sperm using the mean value of 5 randomly selected subsamples [31] (repeatability: *r* ± SE = 0.924 ± 0.005, *p* < .001, *n* = 595 male-days).

When testing sperm count, we also collected 2 samples per male to determine sperm velocity [31]. For each sample, we pipetted a 3-μl solution of 3 sperm bundles and extender medium into the center of a cell in a 12-cell multitest slide (MP Biomedicals, USA) coated with 1% polyvinyl alcohol solution. The sample was activated with 3 μl of 125 mM KCl and 2 mg/ml bovine serum albumin for 30 seconds and covered with a coverslip. We recorded the velocity for 81.94 ± 2.92 *SE* sperm tracks per ejaculate and measured (a) curvilinear velocity (VCL): the actual velocity along the trajectory, (b) average path velocity (VAP): the average velocity over a smoothed cell path and (c) straight-line velocity (VSL) using the CEROS Sperm Tracker. Given that all 3 measures were highly correlated (VCL-VAP: *r* = 0.998; VCL-VSL: *r* = 0.999; VAP-VSL: *r* = 0.999; *n* = 300), we report the actual velocity (VCL).

### Statistical analysis

Our analysis plan was preregistered online (osf.io/swdv7).

#### Preliminary test

Given the sample distribution of each trait, separate generalized linear mixed models (GLMMs) with quasi-Poisson error were used for (a) number of mating attempts and (b) number of nips and approaches to rival, while (c) time spent near the female was analyzed using a linear mixed model (LMM). We considered the state of the gonopodium (ablated, non-ablated), test day (Day 0, Day 7), and standardized SL of focal male (mean = 0, SD = 1) as fixed factors. We included all 2-way interactions (gonopodium state * day, day * focal male size, gonopodium state * focal male size) in our initial models. Male identity was treated as a random factor to account for repeated measurements from each male. We included the standardized size difference between the focal and the new rival male in behavioral test as a covariate because relatively larger males tended to be more aggressive [66]. If gonopodium state and test day showed a significant interaction, we ran separate generalized linear models (GLMs) with negative binomial error for each day to test for differences in behavior between *ablated* and *non-ablated* males.

#### Main experiment

We ran separate LMMs for (a) standard length; (b) body depth; (c) immune response; (d) time spent near female; (e) total sperm count (log-transformed); (f) sperm replenishment rate (log-transformed); and (g) sperm velocity. We ran separate GLMMs with quasi-Poisson error for (h) number of mating attempts (accounting for zero-inflation) and (i) number of nips and rapid approaches. Reproductive treatment (naïve, ablated, non-ablated) and age at testing (week 8, week 16) were considered as fixed factors, and we included their 2-way interaction in initial models. If there was a significant interaction, we ran separate general linear models (LMs) for weeks 8 and 16 to test if the costs of reproduction changed with age at testing.

We included the standardized body size of focal males before the first and second half of the treatment period as a covariate to account for any effect of pretreatment body size on subsequent mating performance and sperm traits (Fig A in S1 Text). We included male identity as a random factor for most traits, except for (b) body depth and (g) sperm velocity, because the variance attributable to male identity was nearly zero in these mixed models. We instead ran simple LMs for these 2 traits. Finally, we included the standardized size difference between the focal and rival male as a covariate for the analyses of mating performance.

In all models, nonsignificant interactions were removed to interpret main effects [67]. We also confirmed that removal of nonsignificant interactions did not significantly lower the model fit (Table F in S1 Text). Tukey’s post hoc tests (*emmeans* package) were run to test for pairwise difference between treatments. We confirmed that the analyses of LMs and LMMs fulfilled model assumptions (i.e., homoscedascity and normally distributed residuals) using Q-Q plots. We conducted dispersion test (*DHARMa* package) to ensure the data variance fulfilled the model assumption in GLMs and GLMMs. Results are presented as mean ± *SE* and the significance level was set at alpha = 0.05 (2-tailed) using the *Anova* function (type III Wald chi-squared tests for GLMs and GLMMs; *F*-tests for LMs and LMMs) in the *car* package of R v4.0.5 [68] using Rstudio v1.3.1093.

## Ethics statement

The project (including fish maintenance, ablation surgery, and trait measurement) received approval from the Australian National University’s Animal Ethics Committee (Ethics Protocol: A2021/04).

## Supporting information

S1 Text Document containing supporting information (Tables A-F and Fig A). (DOCX)

## Abbreviations

BD: body depth
GLM: generalized linear model
GLMM: generalized linear mixed model
LM: linear model
LMM: linear mixed model
SL: standard length

## References

[1] Healy K, Ezard THG, Jones OR, Salguero-Gómez R, Buckley YM. Animal life history is shaped by the pace of life and the distribution of age-specific mortality and reproduction. Nat Ecol Evol. 2019;3:1217–1224. doi: 10.1038/s41559-019-0938-7 31285573

[2] Archer CR, Paniw M, Vega-Trejo R, Sepil I. A sex skew in life-history research: the problem of missing males. Proc R Soc B-Biol Sci. 2022;289:20221117. doi: 10.1098/rspb.2022.1117 35892214 PMC9332873

[3] Lemaître JF, Berger V, Bonenfant C, Douhard M, Gamelon M, Plard F, et al. Early-late life trade-offs and the evolution of ageing in the wild. Proc R Soc B. 2015;282:20150209. doi: 10.1098/rspb.2015.0209 25833848 PMC4426628

[4] Kotiaho JS. Costs of sexual traits: a mismatch between theoretical considerations and empirical evidence. Biol Rev. 2001;76:365–376. doi: 10.1017/s1464793101005711 11569789

[5] Bonduriansky R, Maklakov A, Zajitschek F, Brooks R. Sexual selection, sexual conflict and the evolution of ageing and life span. Funct Ecol. 2008;22:443–453. doi: 10.1111/j.1365-2435.2008.01417.x

[6] Hunt J, Brooks R, Jennions MD, Smith MJ, Bentsen CL, Bussière LF. High-quality male field crickets invest heavily in sexual display but die young. Nature. 2004;432:1024–1027. doi: 10.1038/nature03084 15616562

[7] Kraus C, Eberle M, Kappeler PM. The costs of risky male behaviour: sex differences in seasonal survival in a small sexually monomorphic primate. Proc R Soc B. 2008;275:1635–1644. doi: 10.1098/rspb.2008.0200 18426751 PMC2602817

[8] Papadopoulos NT, Liedo P, Müller HG, Wang JL, Molleman F, Carey JR. Cost of reproduction in male medflies: the primacy of sexual courting in extreme longevity reduction. J Insect Physiol. 2010;56:283–287. doi: 10.1016/j.jinsphys.2009.10.014 19896949 PMC3018851

[9] South SH, Steiner D, Arnqvist G. Male mating costs in a polygynous mosquito with ornaments expressed in both sexes. Proc R Soc B. 2009;276:3671–3678. doi: 10.1098/rspb.2009.0991 19640881 PMC2817312

[10] Emery Thompson M, Georgiev AV. The high price of success: costs of mating effort in male primates. Int J Primatol. 2014;35:609–627. doi: 10.1007/s10764-014-9790-4

[11] Edler R, Goymann W, Schwabl I, Friedl TWP. Experimentally elevated testosterone levels enhance courtship behaviour and territoriality but depress acquired immune response in Red Bishops *Euplectes orix*. Ibis. 2011;153:46–58. doi: 10.1111/j.1474-919X.2010.01075.x

[12] McKean KA, Nunney L. Increased sexual activity reduces male immune function in *Drosophila melanogaster*. Proc Natl Acad Sci U S A. 2001;98:7904–7909. doi: 10.1073/pnas.131216398 11416162 PMC35441

[13] Kelly CD, Jennions MD. Sexual selection and sperm quantity: meta-analyses of strategic ejaculation. Biol Rev. 2011;86:863–884. doi: 10.1111/j.1469-185X.2011.00175.x 21414127

[14] Lüpold S, de Boer RA, Evans JP, Tomkins JL, Fitzpatrick JL. How sperm competition shapes the evolution of testes and sperm: a meta-analysis. Philos Trans R Soc B. 2020;375:20200064. doi: 10.1098/rstb.2020.0064 33070733 PMC7661448

[15] Olsson M, Madsen T, Shine R. Is sperm really so cheap? Costs of reproduction in male adders, *Vipera berus*. Proc R Soc B. 1997;264:455–459. doi: 10.1098/rspb.1997.0065

[16] Lüpold S, Manier MK, Puniamoorthy N, Schoff C, Starmer WT, Luepold SHB, et al. How sexual selection can drive the evolution of costly sperm ornamentation. Nature. 2016;533:535–538. doi: 10.1038/nature18005 27225128

[17] Macartney EL, Crean AJ, Nakagawa S, Bonduriansky R. Effects of nutrient limitation on sperm and seminal fluid: a systematic review and meta-analysis. Biol Rev. 2019;94:1722–1739. doi: 10.1111/brv.12524 31215758

[18] Lemaître JF, Gaillard JM, Ramm SA. The hidden ageing costs of sperm competition. Ecol Lett. 2020;23:1573–1588. doi: 10.1111/ele.13593 32906225

[19] Parker GA. Sperm competition games: raffles and roles. Proc R Soc B. 1990;242:120–126. doi: 10.1098/rspb.1990.0114

[20] Parker GA, Lessells CM, Simmons LW. Sperm competition games: a general model for precopulatory male-male competition. Evolution. 2013;67:95–109. doi: 10.1111/j.1558-5646.2012.01741.x 23289564

[21] Parker GA, Pizzari T. Sperm competition and ejaculate economics. Biol Rev. 2010;85:897–934. doi: 10.1111/j.1469-185X.2010.00140.x 20560928

[22] Lüpold S, Tomkins JL, Simmons LW, Fitzpatrick JL. Female monopolization mediates the relationship between pre- and postcopulatory sexual traits. Nat Commun. 2014;5:3184. doi: 10.1038/ncomms4184 24452310

[23] Lüpold S, Simmons LW, Grueter CC. Sexual ornaments but not weapons trade off against testes size in primates. Proc R Soc B. 2019;286:20182542. doi: 10.1098/rspb.2018.2542 30966988 PMC6501695

[24] Chang C-C, Moiron M, Sanchez-Tójar A, Niemelae PT, Laskowski KL. What is the meta-analytic evidence for life-history trade-offs at the genetic level? Ecol Lett. 2024;27:e14354. doi: 10.1111/ele.14354 38115163

[25] Maklakov AA, Immler S. The expensive germline and the evolution of ageing. Curr Biol. 2016;26:577–586. doi: 10.1016/j.cub.2016.04.012 27404253

[26] Tullis A, Straube CHT. The metabolic cost of carrying a sexually selected trait in the male fiddler crab *Uca pugilator*. J Exp Biol. 2017;220:3641–3648. doi: 10.1242/jeb.163816 28794227

[27] Warburton NM, Bateman PW, Fleming PA. Sexual selection on forelimb muscles of western grey kangaroos (skippy was clearly a female). Biol J Linn Soc. 2013;109:923–931. doi: 10.1111/bij.12090

[28] Simmons LW, Emlen DJ, Tomkins JL. Sperm competition games between sneaks and guards: a comparative analysis using dimorphic male beetles. Evolution. 2007;61:2684–2692. doi: 10.1111/j.1558-5646.2007.00243.x 17941836

[29] Metzler S, Heinze J, Schrempf A. Mating and longevity in ant males. Ecol Evol. 2016;6:8903–8906. doi: 10.1002/ece3.2474 28035278 PMC5192810

[30] Iglesias-Carrasco M, Fox RJ, Vincent A, Head ML, Jennions MD. No evidence that male sexual experience increases mating success in a coercive mating system. Anim Behav. 2019;150:201–208. doi: 10.1016/j.anbehav.2019.02.012

[31] Vega-Trejo R, Fox RJ, Iglesias-Carrasco M, Head ML, Jennions MD. The effects of male age, sperm age and mating history on ejaculate senescence. Funct Ecol. 2019;33:1267–1279. doi: 10.1111/1365-2435.13305

[32] Preston BT, Stevenson IR, Pemberton JM, Wilson K. Dominant rams lose out by sperm depletion. Nature. 2001;409:681–682. doi: 10.1038/35055617 11217847

[33] Chung MHJ, Fox RJ, Jennions MD. Fine-scale genital morphology affects male ejaculation success: an experimental test. Biol Lett. 2020;16:20200251. doi: 10.1098/rsbl.2020.0251 32574532 PMC7336860

[34] Chung MHJ, Jennions MD, Fox RJ. Quantifying the costs of pre- and postcopulatory traits for males: evidence that costs of ejaculation are minor relative to mating effort. Evol Lett. 2021;5:315–327. doi: 10.1002/evl3.228 34367658 PMC8327938

[35] Chung MHJ, Jennions MD, Fox RJ. Novel ablation technique shows no sperm priming response by male eastern mosquitofish to cues of female availability. Behav Ecol Sociobiol. 2019;73:167. doi: 10.1007/s00265-019-2779-4

[36] Wilson RS. Temperature influences the coercive mating and swimming performance of male eastern mosquitofish. Anim Behav. 2005;70:1387–1394. doi: 10.1016/j.anbehav.2004.12.024

[37] Langerhans RB. Genital evolution. In: Evans JP, Pilastro A, Schlupp I, editors. Ecology and evolution of poeciliid fishes. Chicago, IL: University of Chicago Press; 2011. pp. 228–240.

[38] O’Dea RE, Jennions MD, Head ML. Male body size and condition affects sperm number and production rates in mosquitofish, *Gambusia holbrooki*. J Evol Biol. 2014;27:2739–2744. doi: 10.1111/jeb.12534 25403851

[39] Kahn AT, Kokko H, Jennions MD. Adaptive sex allocation in anticipation of changes in offspring mating opportunities. Nat Commun. 2013;4:1603. doi: 10.1038/ncomms2634 23511468

[40] Gasparini C, Devigili A, Pilastro A. Sexual selection and ageing: interplay between pre- and post-copulatory traits senescence in the guppy. Proc R Soc B. 2019;286:20182873. doi: 10.1098/rspb.2018.2873 30963845 PMC6408904

[41] Cattelan S, Evans JP, Pilastro A, Gasparini C. The effect of sperm production and mate availability on patterns of alternative mating tactics in the guppy. Anim Behav. 2016;112:105–110. doi: 10.1016/j.anbehav.2015.11.024

[42] Guevara-Fiore P, Endler JA. Female receptivity affects subsequent mating effort and mate choice in male guppies. Anim Behav. 2018;140:73–79. doi: 10.1016/j.anbehav.2018.04.007

[43] Reznick D. Costs of reproduction: an evaluation of the empirical evidence. Oikos. 1985;44:257–267. doi: 10.2307/3544698

[44] Rowe L, Houle D. The lek paradox and the capture of genetic variance by condition dependent traits. Proc R Soc B. 1996;263:1415–1421. doi: 10.1098/rspb.1996.0207

[45] Simmons LW, Lüpold S, Fitzpatrick JL. Evolutionary trade-off between secondary sexual traits and ejaculates. Trends Ecol Evol. 2017;32:964–976. doi: 10.1016/j.tree.2017.09.011 29050795

[46] Preston BT, Jalme MS, Hingrat Y, Lacroix F, Sorci G. Sexually extravagant males age more rapidly. Ecol Lett. 2011;14:1017–1024. doi: 10.1111/j.1461-0248.2011.01668.x 21806745

[47] Girard MB, Elias DO, Kasumovic MM. Female preference for multi-modal courtship: multiple signals are important for male mating success in peacock spiders. Proc R Soc B. 2015;282:20152222. doi: 10.1098/rspb.2015.2222 26631566 PMC4685782

[48] Devigili A, Belluomo V, Locatello L, Rasotto MB, Pilastro A. Postcopulatory cost of immune system activation in *Poecilia reticulata*. Ethol Ecol Evol. 2017;29:266–279. doi: 10.1080/03949370.2016.1152305

[49] Williams GC. Pleiotropy, natural selection, and the evolution of senescence. Evolution. 1957;11:398–411. doi: 10.1111/j.1558-5646.1957.tb02911.x

[50] Kirkwood TBL. Evolution of aging. Nature. 1977;270:301–304. doi: 10.1038/270301a0 593350

[51] Noble DWA, Lagisz M, O’Dea RE, Nakagawa S. Nonindependence and sensitivity analyses in ecological and evolutionary meta-analyses. Mol Ecol. 2017;26:2410–2425. doi: 10.1111/mec.14031 28133832

[52] Griffiths SW. Sex differences in the trade-off between feeding and mating in the guppy. J Fish Biol. 1996;48:891–898.

[53] McGlothlin JW, Jawor JM, Ketterson ED. Natural variation in a testosterone-mediated trade-off between mating effort and parental effort. Am Nat. 2007;170:864–875. doi: 10.1086/522838 18171169

[54] Gleason ED, Fuxjager MJ, Oyegbile TO, Marler CA. Testosterone release and social context: when it occurs and why. Front Neuroendocrinol. 2009;30:460–469. doi: 10.1016/j.yfrne.2009.04.009 19422843

[55] Buchanan KL, Evans MR, Goldsmith AR, Bryant DM, Rowe LV. Testosterone influences basal metabolic rate in male house sparrows: a new cost of dominance signalling? Proc R Soc B. 2001;268:1337–1344. doi: 10.1098/rspb.2001.1669 11429132 PMC1088746

[56] Powers SK, Jackson MJ. Exercise-induced oxidative stress: cellular mechanisms and impact on muscle force production. Physiol Rev. 2008;88:1243–1276. doi: 10.1152/physrev.00031.2007 18923182 PMC2909187

[57] Mautz BS, Møller AP, Jennions MD. Do male secondary sexual characters signal ejaculate quality? A meta-analysis. Biol Rev. 2013;88:669–682. doi: 10.1111/brv.12022 23374138

[58] Aich U, Head ML, Fox RJ, Jennions MD. Male age alone predicts paternity success under sperm competition when effects of age and past mating effort are experimentally separated. Proc R Soc B. 2021;288:20210979. doi: 10.1098/rspb.2021.0979 34315259 PMC8316792

[59] Nussey DH, Coulson T, Delorme D, Clutton-Brock TH, Pemberton JM, Festa-Bianchet M, et al. Patterns of body mass senescence and selective disappearance differ among three species of free-living ungulates. Ecology. 2011;92:1936–1947. doi: 10.1890/11-0308.1 22073785

[60] Weladji RB, Gaillard JM, Yoccoz NG, Holand Ø, Mysterud A, Loison A, et al. Good reindeer mothers live longer and become better in raising offspring. Proc R Soc B. 2006;273:1239–1244. doi: 10.1098/rspb.2005.3393 16720397 PMC1560281

[61] Vinogradov AE. Male reproductive strategy and decreased longevity. Acta Biotheor. 1998;46:157–160. doi: 10.1023/a:1001181921303 9691260

[62] Clutton-Brock TH, Isvaran K. Sex differences in ageing in natural populations of vertebrates. Proc R Soc B. 2007;274:3097–3104. doi: 10.1098/rspb.2007.1138 17939988 PMC2293943

[63] Abràmoff MD, Magalhães PJ, Ram SJ. Image processing with ImageJ. Biophoton Int. 2004;11:36–42.

[64] Garratt M, Try H, Neyt C, Brooks RC. Exposure to female olfactory cues hastens reproductive ageing and increases mortality when mating in male mice. Proc R Soc B. 2024;291:20231848. doi: 10.1098/rspb.2023.1848 38412966 PMC10898972

[65] Billard R, Puissant C. La spermatogenese de Poecilia reticulata. II. La production spermatogénétique. Ann Biol Anim Biochim Biophys. 1969;9:307–313.

[66] Harrison LM, Vega-Trejo R, Jennions MD. The effect of brief or prolonged bouts of winning or losing male-male contests on plasticity in sexually selected traits. Am Nat. 2023;201:442–459. doi: 10.1086/722829 36848507

[67] Engqvist L. The mistreatment of covariate interaction terms in linear model analyses of behavioural and evolutionary ecology studies. Anim Behav. 2005;70:967–971. doi: 10.1016/j.anbehav.2005.01.016

[68] R Core Team. A language and environment for statistical computing. 2013.

