# Supplementary material for: Male allocation to ejaculation and mating effort imposes different life history trade-offs
Source: PLoS Biol. 2024 May 24;22(5):e3002519. doi: 10.1371/journal.pbio.3002519 (PMC11156437; doi:10.1371/journal.pbio.3002519)
Supplement: S1 Text — (DOCX) [file pbio.3002519.s001.docx]

**S1 Text**

Supporting Information for

**Male allocation to ejaculation and mating effort imposes different life history trade-offs**

Meng-Han Joseph Chung^*^, Rebecca J. Fox, and Michael D. Jennions

*****[chungmenghan@gmail.com](mailto:chungmenghan@gmail.com)

**Table A.** Statistical outputs of initial models including all interaction terms for the surgery effect (i.e., gonopodium state) on mating behaviors in the presence of male rivals. The bold font highlights significance at the 0.05 level.

| **Trait** | **Predictor** | **Test statistic** | **p** |
| --- | --- | --- | --- |
| **Number of nips  and approaches** | Gonopodium state * Day | *χ²*_1_ = 2.886 | .089 |
|  | Body length * Gonopodium state | *χ²*_1_ = 0.360 | .548 |
|  | Body length * Day | *χ²*_1_ = 4.609 | **.032** |
|  | Gonopodium state | *χ²*_1_ = 0.776 | .378 |
|  | Day | *χ²*_1_ = 35.393 | **< .001** |
|  | Body length (standardized) | *χ²*_1_ = 0.096 | .757 |
|  | Male size difference (standardized) | *χ²*_1_ = 8.753 | **.003** |
| **Time spent near female** | Gonopodium state * Day | *F*_1,132.12_ = 0.062 | .804 |
|  | Body length * Gonopodium state | *F*_1,131.78_ = 0.596 | .441 |
|  | Body length * Day | *F*_1,130.08_ = 0.508 | .477 |
|  | Gonopodium state | *F*_1,249.01_ = 0.275 | .600 |
|  | Day | *F*_1,133.01_ = 0.604 | .438 |
|  | Body length (standardized) | *F*_1,220.56_ = 2.804 | .095 |
|  | Male size difference (standardized) | *F*_1,235.93_ = 0.242 | .624 |
| **Number of mating attempts** | Gonopodium state * Day | *χ²*_1_ = 5.988 | **.014** |
|  | Body length * Gonopodium state | *χ²*_1_ = 0.012 | .912 |
|  | Body length * Day | *χ²*_1_ = 2.960 | .085 |
|  | Gonopodium state | *χ²*_1_ = 2.549 | .110 |
|  | Day | *χ²*_1_ = 32.976 | **< .001** |
|  | Body length (standardized) | *χ²*_1_ = 0.718 | .397 |
|  | Male size difference (standardized) | *χ²*_1_ = 1.289 | .256 |

**Table B.** Statistical outputs for interactions and main effects of reproductive treatment and age at testing on somatic traits. Given a significant interaction, the main effects were tested separately at weeks 8 and 16. The bold font highlights significance at the 0.05 level.

| **Trait** | **Predictor** | **Test statistic** | **p** |
| --- | --- | --- | --- |
| ***Standard length*** |  |  |  |
| **Model with interaction** | Reproductive treatment * Age at testing | *F*_2,156.19_ = 24.307 | **< .001** |
|  | Reproductive treatment | *F*_2,301.98_ = 0.756 | .471 |
|  | Age at testing | *F*_1,168.54_ = 164.130 | **< .001** |
|  | Initial body length (standardized) | *F*_1,162.89_ = 8241.10 | **< .001** |
| Week 8 | Reproductive treatment | *F*_2,159_ = 29.208 | **< .001** |
|  | Initial body length (standardized) | *F*_1,159_ = 3460.404 | **< .001** |
| Week 16 | Reproductive treatment | *F*_2,142_ = 1.373 | .257 |
|  | Initial body length (standardized) | *F*_1,142_ = 6098.000 | **< .001** |
| ***Body depth*** |  |  |  |
| **Model with interaction** | Reproductive treatment * Age at testing | *F*_2,302_ = 39.031 | **< .001** |
|  | Reproductive treatment | *F*_2,302_ = 0.627 | .535 |
|  | Age at testing | *F*_1,302_ = 8.643 | **.004** |
|  | Initial body depth (standardized) | *F*_1,302_ = 2352.457 | **< .001** |
| Week 8 | Reproductive treatment | *F*_2,159_ = 80.781 | **< .001** |
|  | Initial body depth (standardized) | *F*_1,159_ = 1139.353 | **< .001** |
| Week 16 | Reproductive treatment | *F*_2,142_ = 0.918 | .402 |
|  | Initial body depth (standardized) | *F*_1,142_ = 1495.706 | **< .001** |
| ***Immune response*** | |  |  |
| **Model with interaction** | Reproductive treatment * Age at testing | *F*_2,149.93_ = 0.622 | .538 |
|  | Reproductive treatment | *F*_2,290.05_ = 1.337 | .264 |
|  | Age at testing | *F*_1,147.54_ = 1.481 | .226 |
|  | Initial body length (standardized) | *F*_1,162.47_ = 0.768 | .382 |
| **Model for main effects** | Reproductive treatment | *F*_2,149.97_ = 4.189 | **.017** |
|  | Age at testing | *F*_1,167.88_ = 0.708 | .401 |
|  | Initial body length (standardized) | *F*_1,163.91_ = 0.727 | .395 |

| **Trait** | | | **Predictor** | **Test statistic** | **p** |
| --- | --- | --- | --- | --- | --- |
| **No. approaches and nips** | | | Age at testing * Reproductive treatment | *χ²*_2_ = 3.597 | .166 |
|  | |  | Age at testing | *χ²*_1_ = 0.010 | .921 |
|  | |  | Reproductive treatment | *χ²*_2_ = 131.841 | **< .001** |
|  | |  | Male size difference (standardized) | *χ²*_1_ = 0.482 | .487 |
|  | |  | Initial body length (standardized) | *χ²*_1_ = 3.738 | .053 |
| **Time spent near female** | | | Age at testing * Reproductive treatment | *F*_2,154.79_ = 2.903 | .058 |
|  | |  | Age at testing | *F*_1,153.04_ = 0.016 | .900 |
|  | |  | Reproductive treatment | *F*_2,297.91_ = 30.565 | **< .001** |
|  | |  | Male size difference (standardized) | *F*_1,300.67_ = 0.774 | .380 |
|  | |  | Initial body length (standardized) | *F*_1,228.44_ = 1.159 | .283 |
| **No. mating attempts** | | |  |  |  |
| Zero-inflation | | | Age at testing * Reproductive treatment | *χ²*_2_ = 1.701 | .427 |
|  |  |  | Age at testing | *χ²*_1_ = 0.032 | .858 |
|  | |  | Reproductive treatment | *χ²*_2_ = 10.130 | **.006** |
|  | |  | Male size difference (standardized) | *χ²*_1_ = 0.009 | .925 |
|  | |  | Initial body length (standardized) | *χ²*_1_ = 0.695 | .404 |
|  | | Conditional | Age at testing * Reproductive treatment | *χ²*_2_ = 3.405 | .182 |
|  | |  | Age at testing | *χ²*_1_ = 0.916 | .339 |
|  | |  | Reproductive treatment | *χ²*_2_ = 25.095 | **< .001** |
|  | |  | Male size difference (standardized) | *χ²*_1_ = 4.788 | **.029** |
|  | |  | Initial body length (standardized) | *χ²*_1_ = 7.618 | **.006** |
| **Total sperm count** | | | Age at testing * Reproductive treatment | *F*_2,147.75_ = 2.809 | .063 |
|  |  | | Age at testing | *F*_1,148.56_ = 0.789 | .376 |
|  |  | | Reproductive treatment | *F*_2,265.43_ = 7.916 | **< .001** |
|  |  | | Initial body length (standardized) | *F*_1,167.20_ = 61.881 | **< .001** |
| **Sperm replenishment rate** | | | Age at testing * Reproductive treatment | *F*_2,146.64_ = 0.293 | .747 |
|  |  |  | Age at testing | *F*_1,145.31_ = 11.024 | **.001** |
|  |  | | Reproductive treatment | *F*_2,275.16_ = 6.021 | **.003** |
|  |  | | Initial body length (standardized) | *F*_1,162.85_ = 37.638 | **< .001** |
| **Sperm velocity (VCL)** | | | Age at testing * Reproductive treatment | *F*_2,293_ = 0.330 | .719 |
|  |  | | Age at testing | *F*_1,293_ = 2.861 | .092 |
|  |  | | Reproductive treatment | *F*_2,293_ = 0.320 | .727 |
|  |  | | Initial body length (standardized) | *F*_1,293_ = 12.981 | **< .001** |

**Table C.** Statistical outputs of initial models including the interaction between reproductive treatment and age at testing on reproductive traits. The bold font highlights significance at the 0.05 level. Final models excluding the non-significant interaction were provided in Table 1 of the main text.

**Cross-study comparison (an additional set of analysis for effect of the presence of rivals)**

To test if interactions with rival males modified the observed effects of pre- and post-copulatory reproductive allocation on trait expression, we compared our findings with those of a previous experiment with a similar design where rivals were absent [1]. We included the presence or absence of male rivals (i.e., study identity) and reproductive treatment as fixed factors, their two-way interaction, and standardized initial standard length (or body depth) as a covariate in the initial models (Table D in S1 Text). If the interaction was significant, we tested for an effect of reproductive treatment in each study separately. We examined all traits that were measured in both studies (see below). The number of mating attempts was analyzed using a generalized linear model with quasi-Poisson error (accounting for zero-inflation), otherwise we ran separate general linear models.

**Reference**

1. Chung MHJ, Jennions MD, Fox RJ. Quantifying the costs of pre- and postcopulatory traits for males: evidence that costs of ejaculation are minor relative to mating effort. Evol Lett. 2021; 5: 315–327. doi: 10.1002/evl3.228.

**Table D.** Statistical outputs of initial models including the interaction between reproductive treatment and the presence of rivals on somatic and reproductive traits. The bold font highlights significance at the 0.05 level. The model of *time spent near female* and final models excluding the non-significant interaction were presented in Table E in S1 Text.

| **Trait** | | | **Predictor** | **Test statistic** | **p** |
| --- | --- | --- | --- | --- | --- |
| **Standard length** | | | Reproductive treatment * Presence of rivals | *F*_2,333_ = 1.695 | .185 |
|  |  |  | Reproductive treatment | *F*_2,333_ = 15.621 | **< .001** |
|  | | | Presence of rivals | *F*_1,333_ = 1.621 | .204 |
|  | | | Initial body length (standardized) | *F*_1,333_ = 5121.400 | **< .001** |
| **Body depth** | | | Reproductive treatment * Presence of rivals | *F*_2,333_ = 0.479 | .620 |
|  |  |  | Reproductive treatment | *F*_2,333_ = 77.996 | **< .001** |
|  | | | Presence of rivals | *F*_1,333_ = 17.271 | **< .001** |
|  | | | Initial body depth (standardized) | *F*_1,333_ = 1436.298 | **< .001** |
| **Immune response** | | | Reproductive treatment * Presence of rivals | *F*_2,321_ = 2.675 | .070 |
|  | | | Reproductive treatment | *F*_2,321_ = 11.492 | **< .001** |
|  | | | Presence of rivals | *F*_1,321_ = 0.298 | .586 |
|  | | | Initial body length (standardized) | *F*_1,321_ = 0.094 | .760 |
| **No. mating attempts** | | |  |  |  |
| Zero-inflation | | | Reproductive treatment * Presence of rivals | *χ²*_2_ = 0.384 | .825 |
|  | |  | Reproductive treatment | *χ²*_2_ = 5.379 | .068 |
|  | |  | Presence of rivals | *χ²*_1_ = 1.735 | .188 |
|  | |  | Initial body length (standardized) | *χ²*_1_ = 0.779 | .377 |
|  | | Conditional | Reproductive treatment * Presence of rivals | *χ²*_2_ = 0.835 | .659 |
|  | |  | Reproductive treatment | *χ²*_2_ = 11.421 | **.003** |
|  | |  | Presence of rivals | *χ²*_1_ = 1.721 | .190 |
|  | |  | Initial body length (standardized) | *χ²*_1_ = 2.111 | .146 |
| **Total sperm count** | | | Reproductive treatment * Presence of rivals | *F*_2,322_ = 0.159 | .853 |
|  |  | | Reproductive treatment | *F*_2,322_ = 13.409 | **< .001** |
|  |  | | Presence of rivals | *F*_1,322_ = 0.546 | .460 |
|  |  | | Initial body length (standardized) | *F*_1,322_ = 82.045 | **< .001** |
| **Sperm replenishment rate** | | | Reproductive treatment * Presence of rivals | *F*_2,316_ = 0.408 | .665 |
|  |  |  | Reproductive treatment | *F*_2,316_ = 3.053 | **.049** |
|  | | | Presence of rivals | *F*_1,316_ = 0.477 | .490 |
|  |  | | Initial body length (standardized) | *F*_1,316_ = 57.654 | **< .001** |
| **Sperm velocity (VCL)** | | | Reproductive treatment * Presence of rivals | *F*_2,323_ = 0.129 | .879 |
|  |  | | Reproductive treatment | *F*_2,323_ = 0.062 | .940 |
|  |  | | Presence of rivals | *F*_1,323_ = 34.340 | **< .001** |
|  |  | | Initial body length (standardized) | *F*_1,323_ = 20.215 | **< .001** |

| **Trait** | | | **Predictor** | **Test statistic** | **p** |
| --- | --- | --- | --- | --- | --- |
| ***Somatic traits*** | | |  |  |  |
| **Standard length** | | | Reproductive treatment | *F*_2,335_ = 41.983 | **< .001** |
|  |  |  | Presence of rivals | *F*_1,335_ = 4.639 | **.032** |
|  |  | | Initial body length (standardized) | *F*_1,335_ = 5117.000 | **< .001** |
| **Body depth** | | | Reproductive treatment | *F*_2,335_ = 150.88 | **< .001** |
|  |  |  | Presence of rivals | *F*_1,335_ = 42.34 | **< .001** |
|  |  | | Initial body depth (standardized) | *F*_1,335_ = 1450.76 | **< .001** |
| **Immune response** | | | Reproductive treatment | *F*_2,323_ = 11.265 | **< .001** |
|  |  | | Presence of rivals | *F*_1,323_ = 2.754 | .098 |
|  |  | | Initial body length (standardized) | *F*_1,323_ = 0.038 | .845 |
| ***Mating performance*** | | | |  |  |
| **No. mating attempts** | | |  |  |  |
| Zero-inflation | | | Reproductive treatment | *χ²*_2_ = 6.938 | **.031** |
|  | |  | Presence of rivals | *χ²*_1_ = 12.509 | **< .001** |
|  | |  | Initial body length (standardized) | *χ²*_1_ = 1.314 | .252 |
|  | | Conditional | Reproductive treatment | *χ²*_2_ = 18.843 | **< .001** |
|  | |  | Presence of rivals | *χ²*_1_ = 0.315 | .575 |
|  | |  | Initial body length (standardized) | *χ²*_1_ = 3.041 | .081 |
| **Time spent near female** | | | Reproductive treatment * Presence of rivals | *F*_2,330_ = 9.526 | **< .001** |
|  |  | | Reproductive treatment | *F*_2,330_ = 0.981 | .376 |
|  |  | | Presence of rivals | *F*_1,330_ = 45.495 | **< .001** |
|  |  | | Initial body length (standardized) | *F*_1,330_ = 2.898 | .090 |
| ***Ejaculate traits*** | | | | | |
| **Total sperm count** | | | Reproductive treatment | *F*_2,324_ = 24.430 | **< .001** |
|  |  | | Presence of rivals | *F*_1,324_ = 0.827 | .364 |
|  |  | | Initial body length (standardized) | *F*_1,324_ = 82.803 | **< .001** |
| **Sperm replenishment rate** | | | Reproductive treatment | *F*_2,318_ = 9.396 | **< .001** |
|  |  |  | Presence of rivals | *F*_1,318_ = 6.144 | **.014** |
|  |  | | Initial body length (standardized) | *F*_1,318_ = 57.479 | **< .001** |
| **Sperm velocity (VCL)** | | | Reproductive treatment | *F*_2,325_ = 0.021 | .979 |
|  |  | | Presence of rivals | *F*_1,325_ = 94.054 | **< .001** |
|  |  | | Initial body length (standardized) | *F*_1,325_ = 20.696 | **< .001** |

**Table E.** Effects of reproductive treatment and the presence of rivals (i.e., levels of male-male competition) on trait expression after 8 weeks. Main effects are obtained from final models excluding non-significant interactions, except for *time spent near female* which had a significant interaction. Statistical outputs of initial models with the interaction are provided in Table D in S1 Text.

**Table F.** Comparison of the best fit of the models.

| *Main experiment* | | | | | | | | |
| --- | --- | --- | --- | --- | --- | --- | --- | --- |
| Trait | Model type | df | AIC | BIC | Log-likelihood | Deviance | *χ²* | p |
| Immune response | Initial | 9 | -650.6 | -617.3 | 334.32 | -668.6 | 1.261 | .532 |
|  | Final | 7 | -653.4 | -627.5 | 333.69 | -667.4 |  |  |
| No. approaches and nips | Initial | 10 | 1600.2 | 1637.5 | -790.07 | 1580.2 | 3.596 | .166 |
|  | Final | 8 | 1599.8 | 1629.6 | -791.87 | 1583.8 |  |  |
| Time spent near female | Initial | 10 | 3781.2 | 3818.5 | -1880.6 | 3761.2 | 5.861 | .053 |
|  | Final | 8 | 3783.0 | 3812.9 | -1883.5 | 3767.0 |  |  |
| No. mating attempts | Initial | 19 | 1822.2 | 1893.2 | -892.11 | 1784.2 | 6.027 | .197 |
|  | Final | 15 | 1820.2 | 1876.2 | -895.12 | 1790.2 |  |  |
| Total sperm count | Initial | 9 | 471.60 | 504.93 | -226.80 | 453.60 | 5.614 | .060 |
|  | Final | 7 | 473.21 | 499.14 | -229.61 | 459.21 |  |  |
| Rate of sperm replenishment | Initial | 9 | 734.03 | 767.21 | -358.02 | 716.03 | 0.597 | .742 |
|  | Final | 7 | 730.63 | 756.44 | -358.31 | 716.63 |  |  |
|  | | Res.df | | RSS | df | Sum of sq | *F* | p |
| Sperm velocity (VCL) | Initial |  | 293 | 200602 |  |  |  |  |
|  | Final |  | 295 | 201054 | -2 | -452.4 | 0.330 | .719 |
| *Cross-study comparison* | | | | | | | | |
| Trait | Model type |  | Res.df | RSS | df | Sum of sq | *F* | p |
| Standard length | Initial |  | 333 | 44.968 |  |  |  |  |
|  | Final |  | 335 | 45.426 | -2 | -0.458 | 1.695 | .185 |
| Body depth | Initial |  | 333 | 7.978 |  |  |  |  |
|  | Final |  | 335 | 8.001 | -2 | -0.023 | 0.479 | .620 |
| Immune response | Initial |  | 321 | 2.846 |  |  |  |  |
|  | Final |  | 323 | 2.893 | -2 | -0.047 | 2.675 | .070 |
| Total sperm count | Initial |  | 322 | 115.17 |  |  |  |  |
|  | Final |  | 324 | 115.28 | -2 | -0.113 | 0.159 | .853 |
| Rate of sperm replenishment | Initial |  | 316 | 241.74 |  |  |  |  |
|  | Final |  | 318 | 242.36 | -2 | -0.625 | 0.408 | .665 |
| Sperm velocity (VCL) | Initial |  | 323 | 150001 |  |  |  |  |
|  | Final |  | 325 | 150121 | -2 | -120.05 | 0.129 | .879 |
|  |  | df | AIC | BIC | Log-likelihood | Deviance | *χ²* | p |
| No. mating attempts | Initial | 15 | 2341.1 | 2398.5 | -1155.5 | 2311.1 | 8.044 | .090 |
|  | Final | 11 | 2341.1 | 2383.2 | -1159.6 | 2319.1 |  |  |

**Fig A. Relationship between initial body length and male reproductive traits.** (A) number of mating attempts (only showing the significant relationship in the conditional part, i.e., exclude zero values; see Table 2 for details); (B) total sperm count; (C) rate of sperm replenishment; (D) sperm velocity. The data and code used to generate this figure can be found in <https://data.mendeley.com/datasets/jv3dyxndtz/1>.
